# Managing and Mitigating the Health Risks of Climate Change: Calling for Evidence-Informed Policy and Action

**DOI:** 10.1289/EHP555

**Published:** 2016-10-01

**Authors:** Shilu Tong, Ulisses Confalonieri, Kristie Ebi, Jorn Olsen

**Affiliations:** 1 School of Public Health and Social Work, Institute of Health and Biomedical Innovation, Queensland University of Technology, Kelvin Grove, Queensland, Australia; 2 Centro de Pesquisas Rene Rachou, Laboratório de Educação em Saúde e Ambiente (LAESA) – Fundação Oswaldo Cruz (FIOCRUZ), Belo Horizonte, Brazil; 3 Department of Global Health, University of Washington, Seattle, Washington, USA; 4 Department of Clinical Epidemiology, Aarhus University Hospital, Aarhus, Denmark

## Abstract

Climate change affects many natural and social systems and processes that are essential for life. It disrupts the Earth’s life-support systems that underpin the world’s capacity to supply adequate food and fresh water, and it disturbs the eco-physical buffering against natural disasters. Epidemiologists need to develop and improve research and monitoring programs to better understand the scale and immediacy of the threat of climate change to human health and to act within a much larger and more comprehensive framework. To address one of the greatest environmental issues of our lifetime, the scientific and policy-making communities should work together to formulate evidence-informed public policy to mitigate greenhouse gas emissions and adapt to its inevitable impacts in this generation and, more importantly, in future generations to come.

## Introduction

The recent United Nations climate treaty achieved unprecedented international support. Representatives from 195 countries met in Paris in December 2015 and agreed to lower their greenhouse gas emissions—an important step towards avoiding many impacts of climate change later this century (Karliner 2015). Climate change affects many natural and social systems and processes necessary for civilization (Pachauri et al. 2014; Patz et al. 2014; Watts et al. 2015). It disrupts the Earth’s life-support systems that underpin the world’s capacity to supply adequate food and fresh water and the eco-physical buffer against natural disasters. These natural systems underlie the attainment and maintenance of good health and well-being in human populations.

Climate change is increasingly recognized as the biggest global health threat (Costello et al. 2009) and opportunity (Watts et al. 2015). Understanding its present and future impacts on population health is of vital scientific and public health importance locally, nationally, and internationally. To date, public concerns and scientific endeavors focused largely on the risks of climate change to economic productivity, livelihoods, tourism, infrastructure, and valued species (McMichael 2012; Smith et al. 2014). For example, pollinators are a key component of global biodiversity, providing vital ecosystem services to crops and wild plants. Global pollinator declines are reported to be associated with habitat loss and fragmentation, agrochemicals, pathogens, alien species, and climate change (Potts et al. 2010). There is now an increasing recognition of its potential risks to human health that are not only related to disasters but also to significant changes in climate systems such as increasing temperatures and altered rainfall patterns. Tackling climate change could be the greatest global health opportunity of this century. Many mitigation and adaptation responses to climate change could lead to direct reductions in the burden of ill health, enhance community resilience, alleviate poverty, and address global inequity (Watts et al. 2015). To address the scale and immediacy of the threat of climate change to human health and well-being, there is an urgent need to develop evidence-informed policy guidelines for action.

The Fifth Assessment Report of the Intergovernmental Panel on Climat**e** Change (IPCC - AR5) concluded that, globally, rising temperatures and changing rainfall patterns were linked to a range of health outcomes (IPCC 2014). Climate change-related alterations in weather and climate patterns would affect many determinants of health (e.g., thermal stress, floods, storms, droughts, bushfires, and food and water quality and quantity), and the geographic range and activity of infectious diseases.

It is important to update the current understanding of the health risks of climate change, to estimate their likely future trajectories, and to provide policy makers with the information needed to take proactive actions to protect individuals, communities, and nations from adverse effects of climate change. This information is within the context of changing population vulnerability. For instance, the world population is projected to reach 9.5 billion by the mid-2050s (mainly due to a growth in the Asian and African populations), and the proportion of the elderly 65 years old and older will more than double (Gerland et al. 2014). It is, therefore, reasonable to anticipate that the impact of climate change on the elderly will be more obvious and become a larger overall disease burden over the coming decades, unless proactive and efficient adaptation measures are implemented.

## New Evidence of Health Effects Attributable to Climate Change and Suboptimal Temperatures

**Figure 1:**
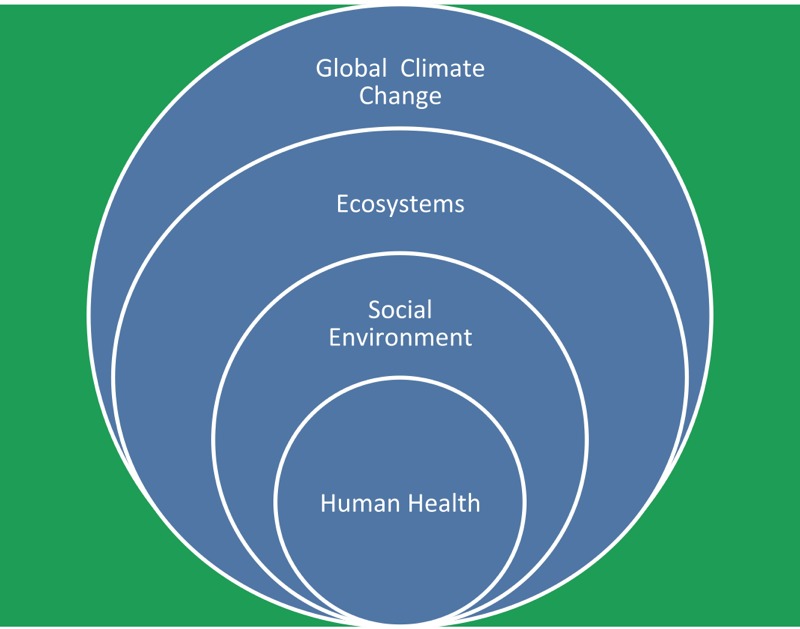
Schematic representation of inter-relations of climate, social environment, ecosystems, and human health. Note: Social environment includes economic development, population aging and growth, globalization, urban planning, medical services, and public health infrastructure; ecosystems include air, water, food, biological agents, land use, and ecosystem services.

Health risks are likely to be influenced by the interaction of future climate change with concomitant changes in sociodemographic, physical, and ecological systems (Figure 1). Global climate change not only affects ecosystems (e.g., changing the range and distribution of vectors), but events attributable to climate change such as heat waves, floods, droughts, and bushfires can influence socioeconomic development (e.g., damaging major roads, community services, and public health infrastructure). All these will have an impact and will increasingly affect human health and sustainable development. Therefore, there is an urgent need to understand these interactions, and facilitate public understanding and informed policy making. However, climatic and non-climatic effects are often complex and difficult to disentangle. The modeling of joint effects is a challenge as there is a wide range of unforeseeable and powerful changes that might occur in social and economic circumstances. It is necessary to develop better research methods (e.g., scenario-based forecasting models) and better data to tackle these complex issues. For example, it is important to project future impacts of climate change on malaria to support effective policy-making and intervention activity concerning malaria control and prevention. Accruing evidence converges on a singular point: The direct effect of climate change on future malaria transmission results in an increase of the simulated length of the malaria transmission season over the tropical highlands (Caminade et al. 2014). This evidence was further corroborated by Siraj et al. (2014) who employed climatic and malaria incidence observations over the plateaus of Ethiopia and Colombia, and is also consistent with results published in the AR5-Working Group II report of the IPCC (IPCC 2014). Climate change will likely expand the distribution of disease vectors to non-endemic areas, increase the length of the disease-transmission season, and facilitate the host–vector-environment interactions in many regions (Smith et al. 2014). The recent spread of the Zika virus may be an indication of what we can expect with continuous environmental changes, and it is suggested that, even though the geographic expansion of the Zika virus is primarily related to increased international travel, climate change may also have helped the spread of this virus (Paz and Semenza 2016). Therefore, it is necessary to enhance the effort to develop vaccines for climate sensitive diseases. It is also important to note that vaccine development would greatly lessen the burden of suffering from those diseases regardless of the climate change impacts.

Another important area of research is to assess the temperature-related mortality and morbidity. An international research team evaluated global variation in the effects of ambient temperature on mortality and found that the temperatures associated with the lowest mortality ranged from the 66th (Taiwan) to the 80th (United Kingdom) percentile (Guo et al. 2014). Although the estimated effects of cold and hot temperatures on mortality varied by community and country, meta-analysis results showed that both cold and hot temperatures increased the risk of mortality in all twelve countries and regions. Cold effects were delayed and lasted for many days, whereas heat effects appeared quickly and did not last long. Guo et al. (2014) concluded that people have some ability to adapt to their local climate type, but both cold and hot temperatures are still associated with increased risk of mortality. Global climate change will not only increase average surface temperature but also aggravate climate variability (IPCC 2013). Evidence suggests that the excess public health risk of climate change stems from both temperature increases and changes of temperature variability (Shi et al. 2015; Vicedo-Cabrera et al. 2016). Therefore, public health strategies need to take both temperature increases and temperature variability into account to alleviate the health impact of ambient temperatures.

A recent study focused on 384 locations in Australia, Brazil, Canada, China, Italy, Japan, South Korea, Spain, Sweden, Taiwan, Thailand, United Kingdom, and the United States, totaling 74,225,200 deaths in different periods between 1985 and 2012 (Gasparrini et al. 2015). In total, 7.71% [95% confidence interval (CI): 7.43%, 7.91%] of mortality was attributed to suboptimal temperature in these countries, with substantial differences between them, from 3.37% in Thailand to 11.47% in Italy. Most of the deaths were probably attributable to cold [7.29% (95% CI: 7.03%, 7.49%)] and heat [0.42% (95% CI: 0.39%, 0.44%)]. Extreme cold and hot temperatures were only responsible for 0.86% (95% CI: 0.84%, 0.87%) of total mortality. Most of the temperature-related mortality burden seemed to be attributable to the contribution of cold. The impact of days of extreme temperature was far less than that attributed to milder, long-lasting but suboptimal weather. These findings are controversial as the data on some confounders (e.g., influenza) were not available. We believe that further research is still required to better understand a net benefit from temperature changes. Despite its limitations, this evidence may have important implications for the planning of public health interventions to minimize the health consequences of adverse temperatures at present, and also for predicting the future impact under climate change scenarios. For example, there is evidence that deaths attributable to cold and hot temperature will substantially shift as climate change proceeds (Huang et al. 2012). Therefore, public health authorities need to prepare for seasonal changes of mortality and significant increases in heat-related deaths in the coming decades.

The World Health Organization (WHO) recently conducted a quantitative risk assessment of effects of climate change on selected causes of death (WHO 2015) and concluded that climate change is expected to cause approximately 250,000 additional deaths per year between 2030 and 2050; of these, 38,000 will be due to heat exposure among the elderly, 48,000 to diarrheal disease, 60,000 to malaria, and 95,000 to undernourished children. These risk estimates may largely be conservative because of the scant number of empirical studies on the health impacts of climate change, particularly from low-income countries (Hosking and Campbell-Lendrum 2012).

## Discussion

Human health is already affected by climate change, and its effects will increase over time. In the longer term, a greater health hazard for many populations will arise from disturbances to environmental, ecological, and social living conditions. As the protection and improvement of human health is one of the ultimate goals of sustainable development, epidemiologists and public health professionals should play an active role in the development of plans for reducing the adverse health effects of climate change at national, regional, and international levels, just like the phasing out of lead from gasoline and taking actions to slow down stratospheric ozone depletion.

Based on the accruing evidence, we make the following policy recommendations:

True primary prevention of adverse climate change impacts on human health (along with many other aspects of the biophysical world) requires a significant worldwide reduction in greenhouse gas emissions, which is possible to achieve with present technology. It is encouraging that the international community recently agreed to curb their greenhouse gas emissions over the coming decades and to limit global temperature rise to < 2° Celsius in this century relative to pre-industrial levels. An international implementation plan on climate policy is urgently needed to ensure sufficient reductions in emissions at the global level. It is important to note that several countries have already substantially invested in renewable energy and much more can be achieved (Frankfurt School-UNEP Collaborating Centre for Climate & Sustainable Energy Finance 2016).
Because additional warming is already committed within the climate system irrespective of actions taken today, cost-effective health-protective adaptive strategies are clearly needed, particularly for vulnerable communities and regions. Adverse environmental and social impacts of climate change will reduce the resilience of many communities, especially the poorest, and in the longer term may overwhelm the coping capacity of many societies (Frumkin et al. 2008; Confalonieri and Tong 2015). Policies and measures that are implemented need to be monitored, evaluated, and refined through well-designed surveillance and research. Little is known about the effective public health adaptation strategies (Toloo et al. 2014). Interdisciplinary research is required to identify, implement, and refine these strategies. More efforts are clearly needed in this area.
Although, at present, most of the temperature-related mortality burden is attributable to cold, and the impact of days of extreme temperature is far less than that attributable to milder but suboptimal weather, this situation can rapidly change as global warming proceeds. Although it is necessary to develop and implement public health interventions for the milder but more significant temperature excursions and increased temperature variability, health authorities should place emphasis on preparing for seasonal changes in mortality patterns and a significant increase in heat-related morbidity and mortality in the near future.
The frequency, intensity, duration, and geographic extent of extreme weather events (e.g., cyclones, heat waves, floods, bushfires, and droughts) are likely to increase in most parts of the world, as climate change continues. Disaster management, community resilience, health care facilities, and public health infrastructure need to be enhanced to respond to these events. Early warning systems for heat waves, floods, and other disasters should be developed and/or improved so that public health impacts of such events can be minimized and prevented.
Vulnerability assessment is still in its infancy, but it should be an area of research that is encouraged to develop. A literature review on this topic is currently underway. It is important to identify and protect vulnerable communities and population groups in the climate change–related health risk assessment and management.
Emerging and resurging infectious diseases are an important health threat. An enhanced effort is required to develop vaccines for climate sensitive diseases. It is also critical to quantify the impact of climate change on the transmission of infectious diseases.
Co-benefit analyses of climate change mitigation and adaptation strategies are an important and emerging field. More evidence-based co-benefit analyses are required from a policy perspective. For example, the phase out of coal is proposed as part of an early and decisive policy package that targets air pollution from the transport, agriculture, and energy sectors, and it aims to reduce the health burden of particulate matter and short-lived climate pollutants, which will yield immediate gains for society (Watts et al. 2015).
It is important to develop scenario-based forecasting models to estimate climate change impacts after incorporating sociodemographic and technological change scenarios because these models can be used in evidence-informed policy making and evaluation. A standard approach for developing scenario-based forecasting models needs to be established and evaluated.
A global health monitoring and surveillance system (e.g., global burden of disease assessment) needs to incorporate climate change impacts (WHO 2014; GBD 2013 Mortality and Causes of Death Collaborators 2015). Health risks posed by climate change are likely to increase and no country will be immune from these risks. Climate resilient health systems are urgently required (WHO 2015).
There is a need to invest in these priority areas from governments, funding agencies, and nongovernment organizations. We believe that these investments are essential to protect population health from the impacts of climate change.

## Conclusion

Epidemiologists need to develop and improve research and monitoring programs to better understand the scale and immediacy of the threat of climate change to human health and act to tackle climate impacts at local, regional, and global levels as these impacts may vary with geographic area and population. Both scientific and policy communities should work together to formulate effective public policy to mitigate greenhouse gas emissions and adapt to its inevitable impacts not only in this generation and, more importantly, in future generations to come. To make this process more effective and efficient, epidemiologists need to embrace policy-oriented research that often requires an interdisciplinary team to address the key research question; researchers should engage decision makers throughout the entire project and to take their feedback seriously when the project evolves; and policy recommendations should be made on the basis of scientific evidence rather than political wills. It is fundamentally important to formulate effective public health policy and adaptation strategies to cope with climate impacts because climate change may be the greatest environmental issue of our lifetime, as well as our children and grandchildren.
